# Volumetric evaluation of osteotomy gap following mandibular bilateral sagittal split osteotomy using a novel semi-automated approach: a pilot study

**DOI:** 10.1007/s00784-024-05753-9

**Published:** 2024-06-06

**Authors:** Kento Odaka, Claudius Steffen, Oliver Wagendorf, Sven Geissler, Tobias Ebker, Kerstin Rubarth, Thanh Thao Nguyen, Emely Lea Bortel, Chompunuch Sarasaen, Georg N. Duda, Max Heiland, Jan Oliver Voss

**Affiliations:** 1 Department of Oral and Maxillofacial Surgery, Charité – Universitätsmedizin Berlin, corporate member of Freie Universität Berlin, Humboldt-Universität zu Berlin, Berlin Institute of Health, Augustenburger Platz 1, 13353 Berlin, Germany; 2 Department of Oral and Maxillofacial Radiology, Tokyo Dental College, 2-9-18, Kandamisaki-Cho, Chiyoda-Ku, Tokyo, 101-0061 Japan; 3 Julius Wolff Institute and Berlin Institute of Health Centre for Regenerative Therapies, Berlin Institute of Health at Charité – Universitätsmedizin, Augustenburger Platz 1, 13353 Berlin, Germany; 4 Institute of Medical Informatics, Charité – Universitätsmedizin Berlin, corporate member of Freie Universität Berlin and Humboldt-Universität zu Berlin, Charitéplatz 1, 10117 Berlin, Germany; 5 Institute of Biometry and Clinical Epidemiology, Charité – Universitätsmedizin Berlin, corporate member of Freie Universität Berlin and Humboldt-Universität zu Berlin, Charitéplatz 1, 10117 Berlin, Germany; 6 XPLORAYTION GmbH, Invalidenstrasse 34, 10115 Berlin, Germany; 7 Berlin Institute of Health (BIH), Anna-Louisa-Karsch-Strasse 2, 10178 Berlin, Germany

**Keywords:** Bilateral sagittal split osteotomy, Volumetric evaluation, Semi-automatic approach, Pseudarthrosis

## Abstract

**Objectives:**

To establish an analysis pipeline for the volumetric evaluation of the osteotomy site after bilateral sagittal split osteotomy (BSSO).

**Patients and methods:**

Cone-beam computed tomography (CBCT) was performed before, directly after BSSO, and 6–12 months after surgery. Image segmentations of each osteotomy gap data set were performed manually by four physicians and were compared to a semi-automatic segmentation approach.

**Results:**

Five patients with a total of ten osteotomy gaps were included. The mean interclass correlation coefficient (ICC) of individual patients was 0.782 and the standard deviation 0.080 when using the manual segmentation approach. However, the mean ICC of the evaluation of anatomical sites and time points separately was 0.214, suggesting a large range of deviation within the manual segmentation of each rater. The standard deviation was 0.355, further highlighting the extent of the variation. In contrast, the semi-automatic approach had a mean ICC of 0.491 and a standard deviation of 0.365, which suggests a relatively higher agreement among the operators compared to the manual segmentation approach. Furthermore, the volume of the osteotomy gap in the semi-automatic approach showed the same tendency in every site as the manual segmentation approach, but with less deviation.

**Conclusion:**

The semi-automatic approach developed in the present study proved to be valid as a standardised method with high repeatability. Such image analysis methods could help to quantify the progression of bone healing after BSSO and beyond, eventually facilitating the earlier identification of patients with retarded healing.

**Supplementary Information:**

The online version contains supplementary material available at 10.1007/s00784-024-05753-9.

## Introduction

Bilateral sagittal split osteotomy (BSSO) is a reliable and common orthognathic procedure for mandibular advancement or setback to treat discrepancy caused by skeletal malocclusion or sleep apnoea [1, 2]. Recently, comprehensive virtual treatment workflows have been demonstrated to achieve high predictability of treatment by using three-dimensional analysis and treatment planning [3]. Bone healing of the osteotomy site can be expected once a stable fixation by osteosynthesis has been achieved [4, 5]. Typically, bone healing occurs through either intramembranous or endochondral bone formation, determined by the osteotomy gap characteristics and fixation technique chosen [6, 7]. Overall, complication rates following BSSO have been reported to be low in retrospective analyses [8–11]. The risk for pseudarthrosis and non-union following BSSO surgery is relatively low, with limited research compared to mandibular fractures [12–16]. In a retrospective study by Ferri et al. [15], pseudarthroses were detected in 0.2% of 5,025 cases. However, if healing does not occur in such cases and non-union persists, the ongoing treatment represents a burden to the patient and the health care system [16–18]. Evaluation of bone healing by radiographic imaging after BSSO is not standardised. However, imaging can aid in detecting any impairment of bone healing, and thus should be considered prior to surgical intervention.

Two-dimensional (2D) imaging, such as a panoramic view, is often used for postoperative evaluation of orthognathic surgery to assess the deviations of the mandibular ramus and detachment or fracture of the plates and screws. Three-dimensional (3D) imaging, such as cone-beam computed tomography (CBCT), can be an effective tool in assessing the morphology of the bone [19–21]. However, whether it could be used in clinical practice to quantify progress in ossification after osteotomy remains so far unknown.

Volumetric analysis of the osteotomy gap after BSSO, as well as of the intersegmental gap between transplanted fibula bone to the mandible, using the reconstructed 3D data generated from manually segmented slices has been recently reported [22, 23]. In these researches, it was demonstrated that the volume of the bony gap can be an important parameter for detecting incomplete bone healing. Although this technique allows the quantitative assessment of ossification after surgery and is also applicable to BSSO cases [24], manual segmentation is very time consuming and the findings strongly influenced by the operator performing the segmentation. A highly automated standard operation procedure for the volumetric evaluation of bone defects is therefore desirable for clinical applications.

The aim of this study was to establish a dedicated volumetric evaluation analysis pipeline to quantify the temporal development of the osteotomy gap in BSSO. Here, we propose a semi-automated analysis pipeline and compare its performance to results obtained through conventional manual segmentation-based analysis.

## Patients and methods

### Ethics

The study was conducted according to the principles outlined in the Declaration of Helsinki. Each patient provided informed consent prior to study inclusion. Ethical approval for data collection and publication was granted by the institutional review board of Charité – Universitätsmedizin Berlin (EA2/107/20).

### Study design

This prospective study investigated two different approaches to evaluating bone healing after mandibular displacement in patients undergoing orthognathic surgery at the Department of Maxillofacial Surgery, Charité – Universitätsmedizin Berlin, Germany. Inclusion criteria included being aged 18 years or above; possessing the capacity to provide informed consent; exhibiting a dentofacial anomaly; and undergoing elective surgical jaw displacement in either the mandible alone or in both the mandible and the maxilla. In total, ten osteotomy gaps in five patients (two female and three male) with a mean age of 31.2 ± 6.53 years were included. The BSSO technique, following the Hunsuck-Epker approach, was exclusively employed for mandibular correction [25]. Internal fixation was accomplished using hand-bent titanium mini-plates with four screw holes and a bar measuring 1 mm in thickness (Gebrüder Martin GmbH & Co. KG, Tuttlingen, Germany). For each site, one plate was used for fixation. Pregnant patients were excluded from the study.

### Image acquisition

CBCT scans were exclusively conducted at the patient’s request for the removal of osteosynthesis plates, adhering to an in-house standard procedure designed to assess for potential bony non-union before proceeding with surgical intervention. Only individuals who had a CBCT immediately after their surgery (baseline) and a subsequent CBCT at least 6 months later (follow-up) were included in the study. All CBCT scans were conducted using the same device (MedSeries H23 from Sophisticated Computertomographic Solutions GmbH, Aschaffenburg, Germany; with a voxel edge length of 400 μm, tube voltage 120 kV, tube current 5 mA). The image data were exported in DICOM format.

### Data analysis

The DICOM data were segmented (1) manually by four independent physicians (authors KO, JOV, OW, and CS) (manual segmentation) and (2) using a newly developed semi-automatic approach (semi-automatic segmentation). For the manual registration, two raters were experienced in the segmentation process (CS and KO) and two raters were unfamiliar with the segmentation process (OW and JOV). The semi-automatic approach was carried out by three operators who had varying levels of experience with segmentation on CT images. For each patient, the jaw was virtually divided into left/ right, lingual/ buccal, and baseline/ follow-up, resulting in eight datasets per patient.

### Manual segmentation

Manual segmentation of the osteotomy gaps was carried out by manually tracing and outlining the gap in slices of “baseline” and “follow-up” using the segmentation editor tools in an image analysis software (3D Slicer, http://www.slicer.org) [26]. The cortical osteotomy gap was defined manually using the freehand selection tool and the interpolation function, individually by each observer. The gap volume for each osteotomy site was subsequently extracted. The methodology employed for assessing gap volume and gap width in this study has been previously described [22–24]. Each clinician was instructed in the labelling task using a standardised protocol prior to the segmentation process. In summary, the cortical bone gap volume and width were examined for each side of the mandible individually, in both the buccal and lingual regions. This allowed for the calculation of total volume (TV) change in a total of four distinct gap measurements per patient. The cancellous bone was not analysed in this study.

### Semi-automatic segmentation

**Fig. 1:**
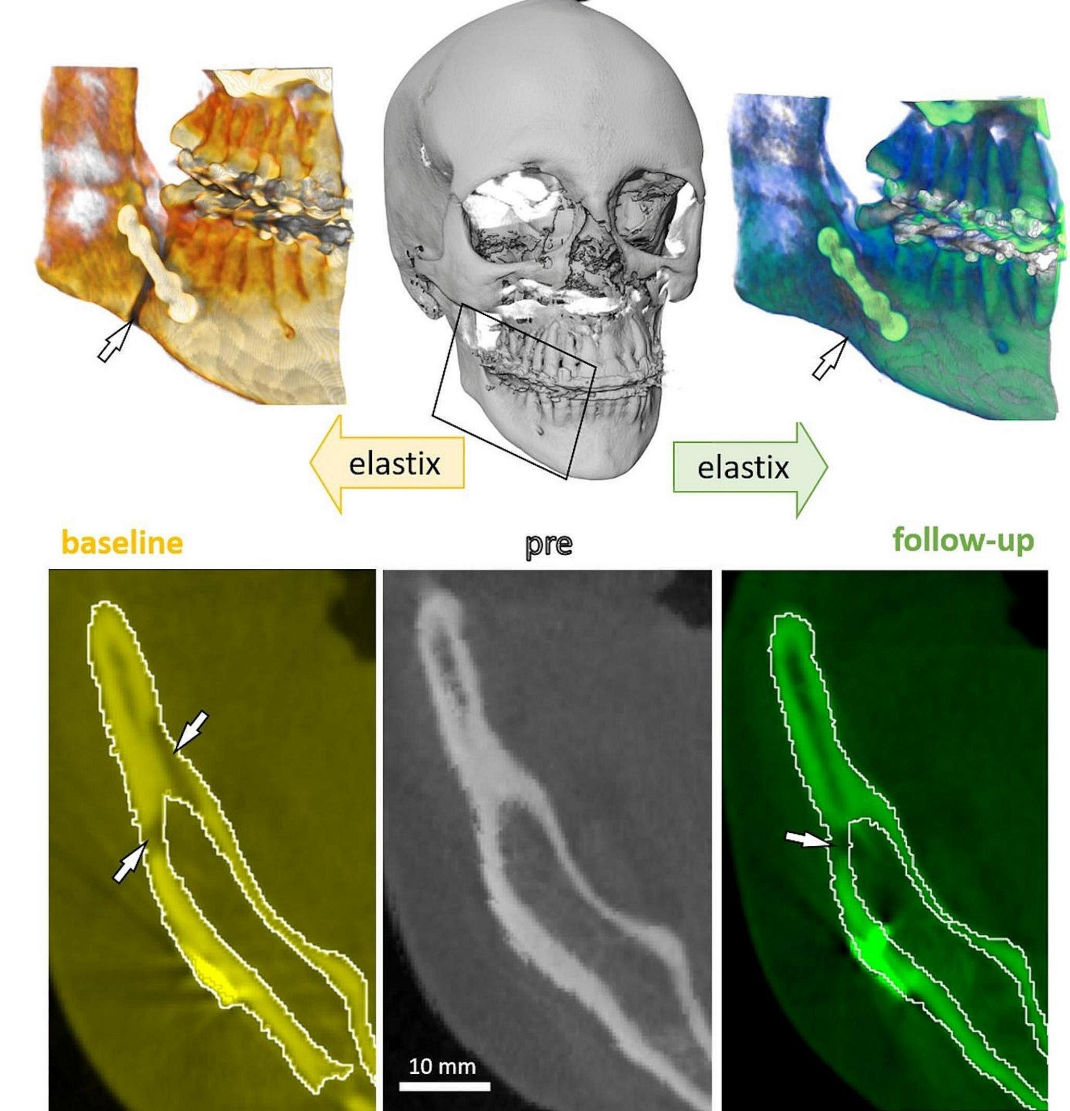
Graphical illustration of the fully automated co-registration. The upper row shows a 3D rendering of pre (no gap) and baseline/ follow-up data. The lower row shows a 2D grayscale overlaid with the white outline from the transformed “pre” dataset and with the “baseline/follow-up” data. The gap is highlighted by white arrows.

The semi-automatic segmentation was divided into two steps. The first step was a fully automated co-registration of the datasets obtained before (pre) and after the osteotomy (baseline, follow-up) (Fig. 1).

The aim of this step was to transform the “pre” dataset so that it enveloped the cortical bone and the gap for the following step. This is shown in Fig. 1, where the white outline of the transformed “pre” dataset is overlaid with the yellow/green “post” data, which includes “baseline” and “follow-up”. The white arrows highlight the gap.

For the registration, all CBCT scans of one patient (preoperative, baseline, follow-up) were first rigidly roughly registered to the same coordinate system using the software simple ITK [27]. The rigid transformation was performed using Euler 3D transformation with a maximum number of 1,000 iterations. Mattes mutual information was selected as a similarity metric, and gradient descent was chosen as an optimiser. Then, the “pre” and “post” CBCT datasets were cropped into the left and right jaw with 192 × 192 × 192 voxels around the gap, yielding six datasets per patient (at three time points, left/ right each). Following this, as shown by Fig. 1, Elastix toolbox was used through an ITK Python interface (“ITK Elastix”), to perform non-rigid image registration between the “pre” and “post” data [27, 28]. The “pre” was set as the moving data to be registered to the corresponding “post” data. B-Spline interpolation of the third order was employed for non-rigid registration with a maximum of 500 iterations and with advanced mattes mutual information as a similarity metric. Adaptive stochastic gradient descent was selected as an optimiser.

**Fig. 2:**
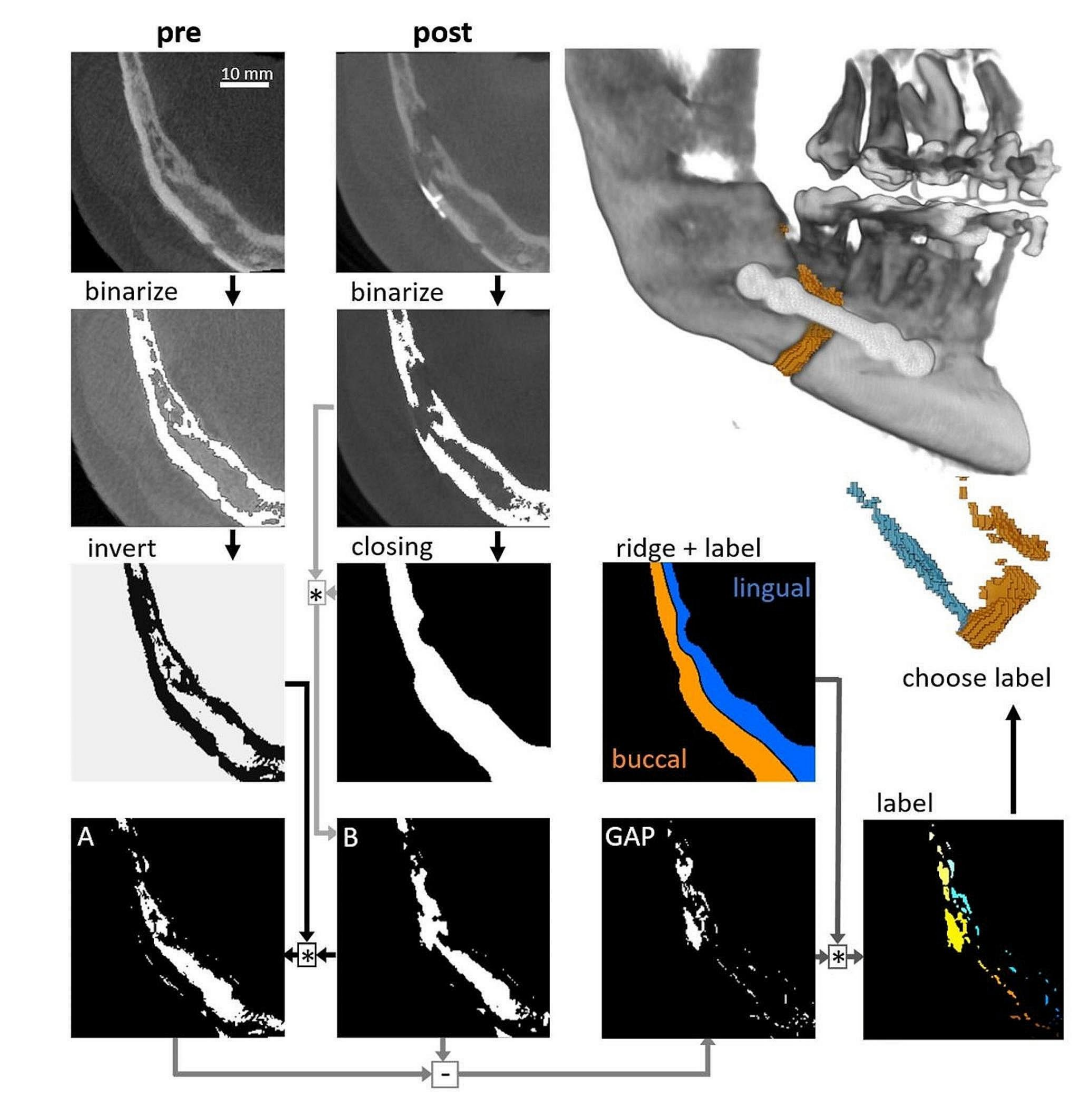
Schematic illustration of the presented semi-automated workflow for segmentation of post-surgical gaps in the jaw. After binarisation of the mineralised bone tissue, the inverted “pre” and the morphologically closed “post” datasets were combined to extract the gap. The gap was divided into lingual and buccal by ridge extraction of the 2D skeleton of the closed ‘post’ dataset. The gap label(s) had to be manually selected in the label image.

The second step was to extract the gap via a workflow including a connected-component analysis. From this analysis, the labels assigned to the gap had to be manually selected. This workflow was implemented using IPSDK (v 3.2, Reactiv’IP, Grenoble, France, 2022; https://www.reactivip.com/) and Python, and is schematically shown in Fig. 2.

Starting with the binarisation of the gray value datasets with a threshold value of 0.75 times, the Otsu-value masks of the “pre” and “post” datasets were generated. The aim was that the “post” mask (including the gap) would be virtually closed by a sphere with a radius of 25 voxels and via morphological combinations with the “pre” mask, the gap could be extracted. To virtually split the result into buccal and lingual, the skeleton of the closed dataset was slice-wise calculated, and the ridge extracted. The morphologically irrelevant parts were removed from the gap mask via connected component labelling, where the gap label had to be chosen manually (Avizo 2019.4, Thermo Fischer Scientific, Waltham Massachusetts). The volume of the gap was then calculated as the sum of all voxels.

### Statistical analysis

All statistical analyses were performed using R Studio (PBC, Boston, USA). Data was presented descriptively by using adequate summary and spread measures such as means with standard deviations or absolute frequencies. To analyse the reliability between the evaluators, two-way interclass correlation coefficients (ICC) were calculated. ICC is a statistical measurement of the agreement or reliability of the ratings. Values close to 1 indicate high agreement and values below 0.5 indicate low agreement [29].

## Results

**Fig. 3:**
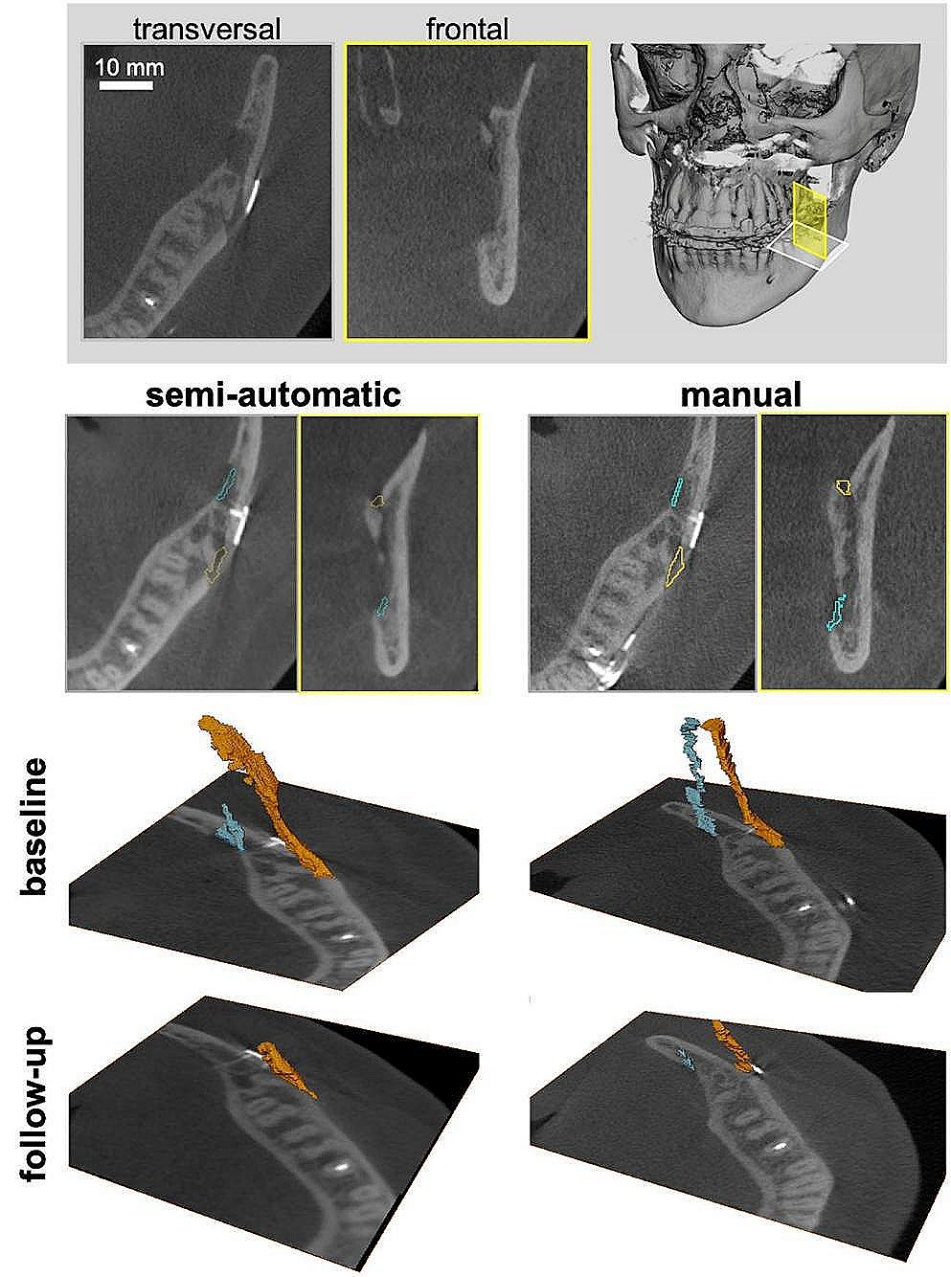
Comparison of the segmentation of the osteotomy gap using the semi-automatic and manual approaches. The buccal gap is shown in orange and the lingual gap in blue. The gaps are superimposed on the gray data in the 2D slices. The transversal (gray) and frontal (yellow) views are shown. The gaps are shown in 3D with the same gray slices for baseline and follow-up.

All patients received BSSO due to dentofacial asymmetry and the surgical procedure was virtually planned preoperatively. Clinical evaluation was uneventful in all five cases. The mean interval between baseline and follow-up imaging was 31.66 ± 2.53 weeks (min = 29; max = 34.72). Segmentation of all ten osteotomy gaps (represented by low-density areas on CBCT imaging) was carried out using both the manual and the semi-automatic approach. The segmentation of the osteotomy gap in both approach is shown in Fig. 3.

Overall manual segmentation by four individual raters showed a relatively low interclass correlation (mean ICC = 0.214, SD = 0.355 over all locations, sites, and time points) without major differences between the buccal (mean ICC = 0.209, SD = 0.430) and lingual (mean ICC = 0.219, SD = 0.329) sites (Supplements 1). However, interclass correlation differed between the time points and the anatomical locations (Table 1). The highest interclass correlation was detected in the segmentation of the left osteotomy of the baseline CBCT (ICC = 0.829). The ICC for the right buccal baseline as well as right buccal follow-ups had negative values (ICC = − 0.047 and ICC = − 0.114, respectively), indicating extremely poor agreement between the raters. In contrast, the overall ICC using the semi-automatic approach (mean ICC = 0.491, SD = 0.365) was higher than the value of the manual approach (Table 2). This suggests that the agreement among the operators using the semi-automatic approach was relatively high.

**Table 1:** Interclass correlation (ICC) for the manual segmentation approach by anatomical site and individual patient

Interclass correlation (ICC) for the manual segmentation approach				
	ICC		95% confidence interval for ICC	
Patient number	Buccal	Lingual	Buccal	Lingual
1	0.655	0.808	0.205 < ICC < 0.968	0.421 < ICC < 0.984
2	0.888	0.768	0.606 < ICC < 0.992	0.350 < ICC < 0.980
3	0.767	0.808	0.357 < ICC < 0.980	0.420 < ICC < 0.984
4	0.755	0.821	0.327 < ICC < 0.979	0.451 < ICC < 0.986
5	0.882	0.669	0.551 < ICC < 0.991	0.205 < ICC < 0.969
Mean	0.789	0.775	0.409 < ICC < 0.982	0.369 < ICC < 0.981
SD	0.098	0.062		
Overall	0.782		0.389 < ICC < 0.982	
SD	0.080			

**Fig. 4:**
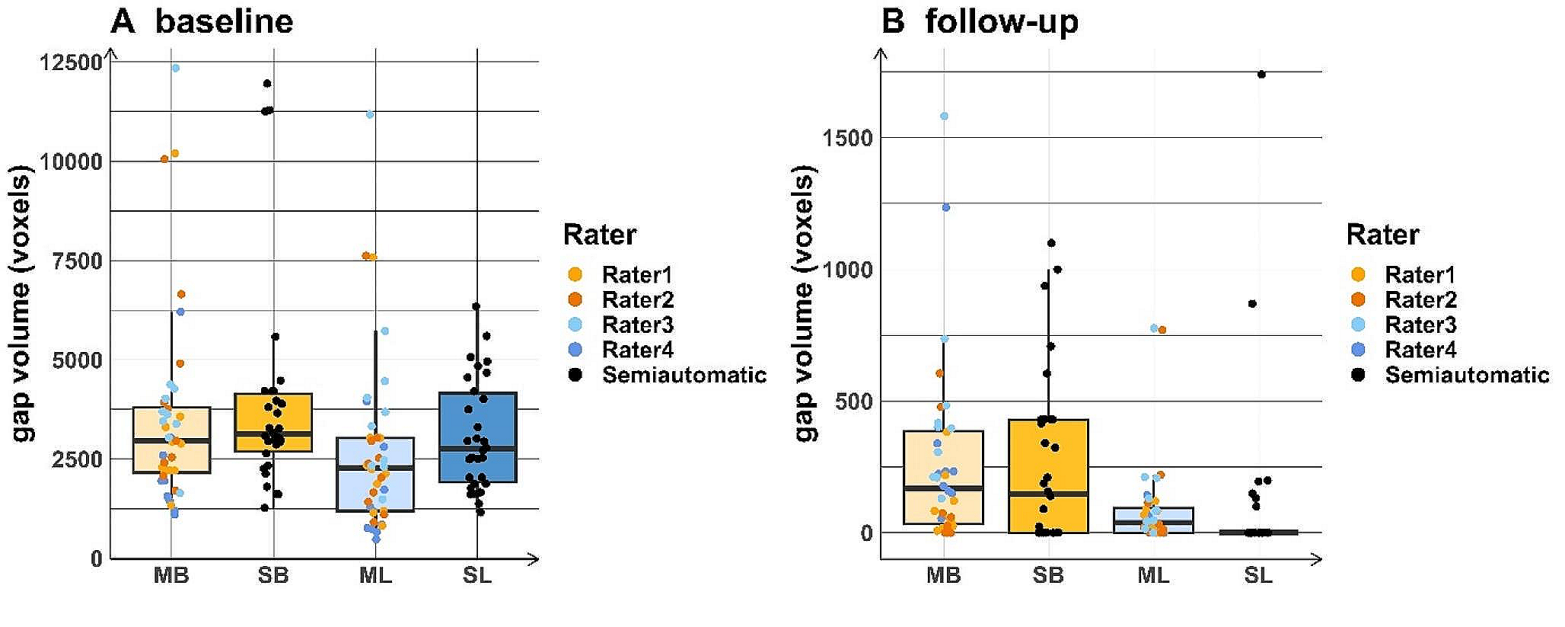
Gap volume distribution by rater

**Fig. 5:**
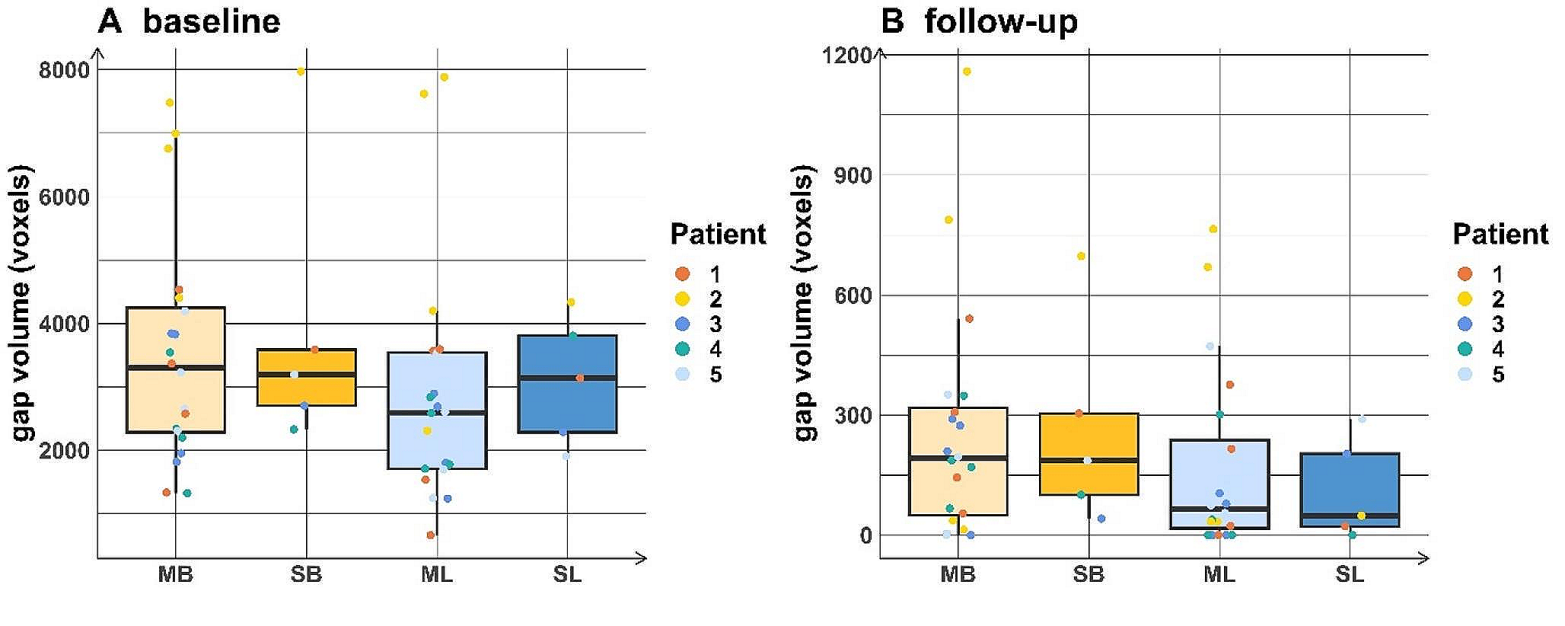
Comparison of the gap volumes between the manual segmentation approach and the semi-automatic approach at baseline (left) as well as at the follow-up time point (right) highlighting each individual patient (Numbers 1–5). Note that the results from the left and right sides are combined here. MB: manual segmentation approach on the buccal side; SB: semi-automatic approach on the buccal side; ML: manual segmentation approach on the lingual side; SL: semi-automatic approach on the lingual side

Table 3 shows the number of voxels in the osteotomy gap of each site in every patient at (A) baseline and (B) follow-up. Figure 4 shows the volume of the osteotomy gap and the comparison between the group of raters and the semi-automatic approach. Figure 5 shows the volume of the osteotomy gap in each individual patient.

The median buccal value was overall greater than the median lingual value both at baseline and on follow-up imaging within both the manual and semi-automatic segmentation approaches. However, while median values were higher from the manual approach compared to the semi-automatic approach at the buccal site on the baseline evaluation, and both sites in the follow-up evaluation, the semi-automatic approach yielded higher median values at the lingual site on the baseline evaluation.

**Table 2:** Interclass correlation for both approaches

Interclass correlation (ICC) for both approaches			
	Buccal	Lingual	Overall
Manual ICC	0.209	0.219	0.214
Manual SD	0.430	0.329	0.355
Semi-automatic ICC	0.703	0.279	0.491
Semi-automatic SD	0.344	0.318	0.365

**Table 3:** The number of voxels in the osteotomy gap

A Baseline				
	**Variable**	**Median**	**Quartile 25**	**Quartile 75**
	Manual buccal	3.300	2.284	4.249
	Manual lingual	2.597	1.708	3.539
	Semi-automatic buccal	3.194	2.705	3.587
	Semi-automatic lingual	3.139	2.288	3.810
B Follow-up				
	**Variable**	**Median**	**Quartile 25**	**Quartile 75**
	Manual buccal	192	50	318
	Manual lingual	65	17	237
	Semi-automatic buccal	186	101	304
	Semi-automatic lingual	49	22	203

The agreement regarding the evaluation of the osteotomy gap differed between the patients and the localisation of the different time points (Table 1). The best overall agreement is seen in patient number 2.

Overall, the semi-automatic approach was far less time consuming than the manual approach. In the manual approach, the time for segmentation differed between every case and each rater, and was influenced by the number of evaluated slices. However, the manual segmentation process was less time consuming with increased experience of the rater. Many factors influencing the time required for the segmentation process in the manual segmentation approach, including experience level of the rater; number of slides; volume of osteotomy gap/ clarity of osteotomy gap; degree of ossification; and the exact time of the segmentation process were not measured as part of the analysis.

Comparison between the gap volumes obtained by the manual segmentation approach and the semi-automatic approach at baseline (left) as well as at the follow-up time point (right) highlighting each individual rater (No. 1–4); raters 1 and 2 were considered to be more experienced than raters 3 and 4. Note that data from the left and right sides are pooled here.

MB: manual segmentation approach on the buccal side; SB: semi-automatic approach on the buccal side; ML: manual segmentation approach on the lingual side; SL: semi-automatic approach on the lingual side.

## Discussion

Bone healing of the osteotomy site after BSSO is an important factor to be assessed in the postoperative follow-up period, but there is no standardised method for the evaluation. The quantitative evaluation method for osteotomy developed in this study may be useful in accurately assessing the osteotomy site and determining treatment plans during follow-up.

Although non-union and pseudarthrosis are generally rare after BSSO, there is a risk that this complication is not recognised in the follow-up period and removal of the osteosynthesis material not attempted. This could result in revision surgery with replacement of the osteosynthesis material and, if necessary, bone grafting. So far, there has been no general recommendation with regard to required radiological imaging, or how this imaging should be interpreted to evaluate bone healing of the osteotomy gap after BSSO surgery. To obtain a stable evaluation of ossification after BSSO, a quantitative assessment of changes occurring locally at the osteotomy site would be an effective approach.

In this study, volumetric evaluation of the change of the osteotomy gap was performed using CBCT. Evaluating the osteotomy site using CBCT after BSSO surgery is considered to be reasonable in the context of radiation exposure and diagnostic usefulness, particularly in high-risk patients. Heo et al. [30] investigated the osteotomy area by using processed images based on panoramic tomography and related it to the bone healing process. The radiopacity of the osteotomy area is related to the distribution of callus mineralisation during the bone healing process [4, 6]. Bone mineral density is strongly related to bone strength: it is therefore suggested that segmenting the newly formed tissue in the gap region represents not only the degree of calcification but also the biomechanical relevance of the tissue [31]. Overall, the mean osteotomy gap on the buccal side was larger than on the lingual side using both approaches in the present study, suggesting wider cortical bone thickness on the buccal side. In addition, the median value at follow-up was lower than the baseline value, particularly on the lingual side, suggesting a reduction of the initial osteotomy gap during bone healing.

In the manual segmentation approach of the present study, four observers performed region extraction based on CBCT slice images. The agreement among the observers depended on the patient, localisation, and time point. Interestingly, the ICC was very low when rating each anatomical location separately, indicating a wide variation between the raters in the quantitative evaluation of the same datasets of post-operative CBCTs (Supplements 1). However, the agreement between the raters is higher when rating each patient separately (Table 1).

The high ICCs when considering locations for individual patients suggest that the locations exhibit some degree of consistency in the assessments across different patients, i.e. the assessments at different locations for the same patient tend to be more similar, indicating that the raters have a good agreement when it comes to patient assessments. Conversely, the low ICC when considering patients for individual locations indicates that there is more variability in patient assessments at the same location. This could be due to variation in patient attributes, making it difficult for raters to reach a consensus in their assessments.

Joskowicz et al. [32] reported that the inter-observer variability in manual delineations for different structures and observers is large and spans a wide range. They suggest that the repeatability of the quantitative evaluations using the manual approach is not high because of the inter-observer variability.

In our study, the morphology of the extracted regions varies with the diverse clinical experiences of the four observers. In the follow-up dataset in particular, the difference in the gap between those determined to have completed healing and those determined to be in the healing process may reflect variation in the gap volume. In addition, the time required for segmentation varied greatly depending on the case and practitioner, and changed during the progress of segmentation (the learning process). Multiple repetitions may reduce the time required for manual segmentation in one patient, but not in another, as the time required for segmentation is influenced by the practitionerr’s recognition of bone boundaries and gaps. While more time was required in cases with extensive bone defects spanning multiple slices, the evaluation and subsequently the segmentation of these defects appeared to be easier than the evaluation of smaller defects.

In the semi-automatic approach group, the osteotomy gap was generated by subtracting the gap and the non-rigid registered “pre” image, which was created based on the preoperative image. Following this, the connected component labels that corresponded to the gap were selected individually. The results showed the same tendency as the manual segmentation group, indicating that this approach can be an effective alternative to manual segmentation. The semi-automatic approach may be a more standardised and reproducible method because the processing time is constant regardless of the difficulty of each case and the expertise level of the operators performing this semi-automatic approach. Furthermore, the segmented results obtained through this approach have a lower standard deviation. However, the variation in the semi-automatic approach may occur in the second step in the workflow to manually extract the connected component. The shape of the chosen segment may affect the value of the osteotomy gap and the deviation. To improve accurate analysis of the osteotomy gap, close communication between the practitioner and the proposed pipeline operators would be required to share their recognition of the region of interest in the surgery. Further investigation is also needed to produce a more reproducible pipeline.

Recently, the computer-assisted approach to evaluate heterogeneity based on medical images, or radiomics, has been developed to aid clinical decision-making and prognosis prediction [33]. Segmentation using a deep learning system is a probability calculation based on density morphological information, and it is easy to separate calcified tissue from non-calcified tissue, even in CBCT images [34]. However, there are areas near an osteotomy that exhibit low density on CBCT after BSSO, such as the mental foramen and mandibular foramen. In the present study, the semi-automatic method was adopted in which the gap was selected manually as connected components to prevent the risk of extracting such structures accidentally and interpreting them to be the osteotomy gap.

This study has several limitations: it couldn’t test for the accuracy of each segmentation method, as the true volume of the osteotomy gap (at baseline and at follow-up) is unknown. However, the aim of the study was to provide an analysis pipeline including the segmentation of the osteotomy gap to evaluate bone healing over time using a semi-automatic approach as a more standardised alternative to the manual approach, prior to a fully automatic approach. To implement a fully automatic segmentation approach in the future, further verification including data accumulation and validity verification will be required.

## Conclusion

This study demonstrated a novel semi-automatic analysis pipeline approach for the volumetric assessment of bone healing after BSSO surgery. This semi-automatic method allows the calculation of bone volume changes with relatively high repeatability when compared to manual segmentation. The presented approach could be helpful clinically to identify and quantify the progress of bone formation in a reliable and robust way to aid in earlier identification of patients at high risk of retarded or non-healing following BSSO surgery, or even in other contexts such as fractures.

### Electronic supplementary material

Below is the link to the electronic supplementary material.

Supplementary Material 1

### Electronic supplementary material

Below is the link to the electronic supplementary material.

Supplementary Material 2

## Data Availability

No datasets were generated or analysed during the current study.
