# Supplementary material for: Volumetric evaluation of osteotomy gap following mandibular bilateral sagittal split osteotomy using a novel semi-automated approach: a pilot study
Source: Clin Oral Investig. 2024 Jun 6;28(7):358. doi: 10.1007/s00784-024-05753-9 (PMC11156743; doi:10.1007/s00784-024-05753-9)
Supplement: Supplementary file 2 — Supplementary Material 2 [file 784_2024_5753_MOESM2_ESM.docx]

| **Interclass correlation (ICC) for the manual segmentation approach** | | | |
| --- | --- | --- | --- |
|  |  | ICC | 95% confidence interval for  ICC population values |
| Right buccal baseline | | -0.047 | −0.202 < ICC < 0.545 |
| Left buccal baseline | | 0.829 | 0.437 < ICC < 0.978 |
| Right lingual baseline | | 0.701 | 0.253 < ICC < 0.957 |
| Left lingual baseline | | 0.688 | 0.284 < ICC < 0.955 |
| Right buccal follow-up | | -0.114 | −0.215 < ICC < 0.378 |
| Left buccal follow-up | | 0.169 | −0.116 < ICC < 0.772 |
| Right lingual follow-up | | 0.015 | −0.210 < ICC < 0.661 |
| Left lingual follow-up | | 0.005 | −0.236 < ICC < 0.668 |
| Buccal Mean | | 0.209 | −0.024 < ICC < 0.668 |
| Buccal SD | | 0.430 |  |
| Lingual Mean | | 0.219 | −0.066 < ICC < 0.755 |
| Lingual SD | | 0.329 |  |
| Overall Mean |  | 0.214 | −0.045 < ICC < 0.712 |
| Overall SD | | 0.355 |  |

**Supplements 1**  Interclass correlation (ICC) in manual segmentation approach with regards to anatomical site and time point
